# Comparative linkage mapping of diploid, tetraploid, and hexaploid *Avena* species suggests extensive chromosome rearrangement in ancestral diploids

**DOI:** 10.1038/s41598-019-48639-7

**Published:** 2019-08-23

**Authors:** Robert G. Latta, Wubishet A. Bekele, Charlene P. Wight, Nicholas A. Tinker

**Affiliations:** 1 Dept. of Biology, Dalhousie University, 1355 Oxford St., Halifax, NS B3H 4R2 Canada; 2 Ottawa Research and Development Centre, Agriculture & Agri-Food Canada, 960 Carling Ave., Ottawa, Ontario K1A 0C6 Canada

**Keywords:** Agricultural genetics, Evolutionary biology

## Abstract

The genus *Avena* (oats) contains diploid, tetraploid and hexaploid species that evolved through hybridization and polyploidization. Four genome types (named A through D) are generally recognized. We used GBS markers to construct linkage maps of A genome diploid (*Avena strigosa* x *A*. *wiestii*, 2n = 14), and AB genome tetraploid (*A*. *barbata* 2n = 28) oats. These maps greatly improve coverage from older marker systems. Seven linkage groups in the tetraploid showed much stronger homology and synteny with the A genome diploids than did the other seven, implying an allopolyploid hybrid origin of *A*. *barbata* from distinct A and B genome diploid ancestors. Inferred homeologies within *A*. *barbata* revealed that the A and B genomes are differentiated by several translocations between chromosomes within each subgenome. However, no translocation exchanges were observed between A and B genomes. Comparison to a consensus map of ACD hexaploid *A*. *sativa* (2n = 42) revealed that the A and D genomes of *A*. *sativa* show parallel rearrangements when compared to the A genomes of the diploids and tetraploids. While intergenomic translocations are well known in polyploid *Avena*, our results are most parsimoniously explained if translocations also occurred in the A, B and D genome diploid ancestors of polyploid *Avena*.

## Introduction

Among its many evolutionary consequences^1–3^, polyploidy is widely recognized as a driver of genome rearrangement^4–6^. This is primarily due to the presence of homeologous chromosomes, which are prone to mispairing at meiosis^7,8^. Crossovers between homeologs can create translocations of medium-to-large chromosomal segments, while gene conversion events can transfer smaller sequences from one homeolog to the other^4^. Numerous examples of intergenomic rearrangement in polyploids have been described^7–11^. Such rearrangements are thought to be less common in diploids, because meiotic mispairing is less likely to occur. Indeed, paleopolyploidy, and subsequent gradual reversion to diploidy, has recently become implicated in fostering genome reshuffling among lineages that are now considered diploid^3,6,12,13^. Nevertheless, translocations between non-homeologous chromosomes in the absence of polyploidy are also known, though even these are sometimes associated with homoploid hybridization – *i*.*e*., hybridization without polyploidization^14,15^.

The oats (*Avena* spp.) form a genus of diploid, tetraploid, and hexaploid grasses with a base chromosome number of seven^16–18^. Most are self-pollinating annuals. Like wheat, the *Avena* polyploids exhibit disomic inheritance and are often designated as allotetraploid or allohexaploid^18,19^. Four cytogenetically distinct genomes, designated A through D, are recognized^16,18^. However, the B and D genomes are known only from polyploid species, since no extant diploids with these genomes have been described.

The most widely cultivated oat species, *A*. *sativa*, is hexaploid and contains the genomes A, C, and D, as do the wild hexaploid oats. A well supported consensus genetic linkage map is available for this economically important species^20^ which maps linkage groups to physical chromosomes. However, separate mapping populations of *A*. *sativa* contain unique rearrangements between homeologous chromosomes, indicating ongoing genomic exchange in this hexaploid. Seven linkage groups share markers primarily with A-genome diploids, while seven others share markers primarily with the C genome diploids^21^. The remaining seven share markers with the AC(DC) tetraploids *A*. *maroccana* (=*A*. *magna*), *A*. *murphyi*, and *A*. *insularis*, which are thus the most likely ancestor(s) of the C and D genomes in hexaploid oat. Furthermore, through map-based haplotype complementation^21^, as well as through previous cytogenetic analysis (summarized by Sanz *et al*.^22^), it appears that translocations between subgenomes within hexaploid *A*. *sativa* are more prevalent between C and D chromosomes than between A and D chromosomes. The above results support a hypothesis whereby the C and D genomes co-existed in a tetraploid oat (likely *A*. *insularis*) prior to hybridization(s) with an A-genome diploid to form the ACD genome hexaploids^21^. Although patterns of homeology in *A*. *sativa* have been heavily disrupted by translocations, four triplets of A/C/D linkage groups appear to have retained substantial linear homeology^20^.

The A and C genome diploids are distinct evolutionary lineages, appearing as separate clades in phylogenies of the diploid *Avena* species^23–26^. Several sub-types have been designated within each group based on karyotype analysis and chromosome pairing^16,18^. The most common subtype among the A-genome diploids is the A_s_ genome subtype, which also shows the strongest cytogenetic similarity to the A genome of the hexaploid *A*. *sativa*^21^. Thus the most intensive study of A genome diploids has been focused on species with this genome subtype, and several mapping populations have been studied^27–29^. It remains unclear which C-genome diploid is the progenitor of *A*. *sativa*, and we know of no genetic mapping population in C genome diploids.

The B-genome of *Avena* is known only from four AB tetraploid species, the most abundant of which is *A*. *barbata*. There has been a long running debate over whether the B genome should be recognized as distinct from the A genome^16,19^, and by extension whether *A*. *barbata* is auto- or allo tetraploid^30^. Early chromosome studies identified partial homologous pairing of A and B genome chromosomes during meiosis in hybrids, although this often occurred in chains, which suggested rearrangement of the A vs B genomes^30–33^. While Southern blot experiments failed to discriminate the genomes^34^, FISH probes specific to the A genome were able to distinguish A and B genomes within *A*. *barbata*^35,36^. Most recently, Chew *et al*.^37^ argued against the recognition of a distinct B genome because multivariate clustering of GBS markers placed AB genome tetraploids near A_s_ genome diploids. However, a more distinct separation of AB tetraploids from the A-genome diploids is obtained by selecting GBS markers in a way that would avoid an ascertainment bias caused by a prevalence of A-genomes in the test population^21^. In any case, the tetraploid *A*. *barbata* exhibits disomic inheritance^30,38^, allowing construction of a partial linkage map using AFLP markers^39^. However, this map is clearly incomplete, since the total length is shorter than most maps of diploid oats^29^, and because it contains nineteen linkage groups for *A*. *barbata’s* 14 chromosomes.

In the present study we had three goals. First, we used Genotyping by Sequencing (GBS) markers to update the linkage maps from *A*. *strigosa* C.I. 3815 × *A*. *wiestii* C.I. 1994^29^ and *A*. *barbata*^39^. Second, we examined clusters of similar loci that segregate within *A*. *barbata* to identify homeologous regions^40^ within this tetraploid, and determine whether the A and B subgenomes are distinct. Third, we identified markers segregating in both mapping populations as anchors to align homologous regions of the diploid and tetraploid genomes, and then extended this to GBS markers on the domestic *A*. *sativa* linkage map. This provides a comparative map across three ploidy levels each containing a copy of the A genome.

## Methods

### Crosses

We used the *A*. *strigosa* C.I. 3815 × *A*. *wiestii* C.I. 1994 F_6:8_ (henceforth SW) recombinant inbred line (RIL) population^28^. Seeds from 96 of the RILs, along with the parents, were obtained from Iowa State University and used for this study.

We also used an existing *A*. *barbata* mapping population^39^, derived from a cross between Californian ecotypes associated with moist and dry habitats^41^. Of the initial 196 RILs, 180 F7 lines were available for GBS. In addition, we genotyped several accessions of the mesic (moist), and xeric (dry) ecotypes, with one accession of each ecotype subjected to a ‘deep read’, so as to capture as many segregating alleles as possible and correctly assign polarity in the cross.

### Genotyping by sequencing

Fresh tissue was grown from seed in the greenhouse (*A*. *barbata*) or growth cabinets (SW). Leaves were dried on silica, and DNA was extracted using QIAGEN DNEasy Mini kits. All RILs of both mapping populations were genotyped using a double restriction digest (*Pst*I and *Msp*I) GBS protocol^42^. Barcoded samples were pooled into 96-plex libraries and subjected to 100 bp single read runs (one per population) on a HiSeq2500 sequencing system. In addition, the *A*. *barbata* mapping population was also subjected to GBS by a single-digest protocol^43^ using *Pst*I alone.

GBS analysis was performed using the Haplotag pipeline^44^ which is specifically designed to address problems of paralogous loci in polyploid species. Haplotag treats tag-level haplotypes, which may differ at multiple SNPs, as discrete alleles, ignoring the potential for very rare recombinations within their 64-base length. It first clusters similar tags based on nearest-neighbor joining of all tags that differ by three or fewer SNPs. It then screens all possible subsets of tags within each cluster to identify putative loci, subject to a set of population-based filtering criteria, then partitions tag-level haplotypes into the best available model of mutually-exclusive loci. We set the filtering criteria such that the maximum heterozygosity was limited to 15%, and that no more than 2 alleles could segregate at a locus within the same RIL population. However, since segregation distortion has been documented in the *A*. *barbata* map^39^, we filtered leniently for allele frequency departures from the expected 1:1 ratio.

### Map Construction and error checking

We constructed framework maps from the most reliable subset of the loci identified by Haplotag. We included only those loci that were scored in >90% of the RILs, with <5% heterozygosity, and with no more than three base pairs difference between alleles. In *A*. *barbata*, loci were included in map construction only if they showed fixed differences between the parental accessions with no missing parental data. In the SW population, marker phase was sometimes ambiguous due to missing parental genotypes and/or parental heterogeneity. Thus, an in-house script was used to phase the markers based on their patterns of recombination in this population. Loci that were identified by Haplotag, but which did not meet the above criteria, were set aside as ‘secondary loci’, and placed on the map after framework construction.

We used MSTMap^45^ to infer the map, using the Kosambi distance function and estimating the recombination distance from a simple count of the number of crossovers. We discarded any linkage group shorter than 15 cM or with fewer than four markers. The probability threshold (*e*) for joining markers into a linkage group was decreased progressively in successive runs until the correct number of linkage groups was obtained (Supplementary Methods). Mis-identified genotypes can inflate map distances by appearing as double recombinants. We therefore screened the linkage map for such errors by removing any apparent double crossover involving fewer than three markers and less than 2 cM. Such genotype calls were converted to missing data (since they were likely in error) and the map was re-estimated (Supplementary Methods).

### Presence-absence variants

To increase the number of mapped loci which might permit us to align the genomes, we screened the data for presence-absence variants (PAV’s). These came from two sources: (a) after loci had been identified by Haplotag, any tags that were ‘left over’ in the tag clusters were treated as potential PAVs (Supplementary Methods), and (b) the large number of ‘singleton’ tags which showed no sequence similarity to any other tags in the data set. PAVs from either source can increase the number of comparisons between mapping populations, but only the former could be used to identify paralogs/homeologs within genomes.

We filtered these tags stringently to identify high-quality PAVs. First, only those which showed fixed differences between the parents were retained. PAV’s needed to be present at >10 read depth in one of the parental accessions (and in the case of *A*. *barbata*, to be present in at least 3/5 accessions of one parent, and absent from all accessions of the other parent). Assuming a high degree of homozygosity in the inbred lines, Mendelian PAVs should be present in 50% of the lines. We therefore eliminated any tag present in fewer than 40% or more than 60% of the RILs, although this prevented identifying any PAVs in regions of segregation distortion.

We placed secondary loci and PAVs on the map at the point of lowest recombination fraction (Supplementary Methods). Because less stringently filtered loci and PAVs are likely to contain more error than the stringently filtered loci used to construct the map, we did not integrate these loci into the map, but any that showed <10% recombination to the closest marker were assigned to that position. These approximate map positions are probably accurate enough to inform our inferences of synteny within and across the linkage maps, while the additional markers increase our power to detect patterns. RFLP markers^29^ and AFLP markers^39^ were also positioned in this way, such that the new maps could be aligned with the original maps.

### Homologs, paralogs and homeologs

To identify homologous tags, we used BLAST^46^ to find the closest sequence match of each tag between the set of mapped tags in *A*. *barbata* and SW. We analyzed homology at the tag (rather than locus) level, since most biallelic loci matched at only one allele, and because PAVs were included (*e*.*g*., similar PAVs segregating in both maps, or a PAV in one map matching an allele at a biallelic locus in the other). We extended this comparison to the GBS map of *A*. *sativa*^20,47^. Each tag was included in only that pair with the closest match, and we excluded tag pairs with more than three base pair mismatches (since this was the threshold for clustering tags in the Haplotag pipeline).

Paralogs and homeologs within *A*. *barbata* were identified in the Haplotag pipeline by cases where more than one segregating locus occurs within a cluster of tags (Supplementary Methods). If more than two segregating loci occurred in a tag cluster, two of those loci were chosen at random. These loci were then mapped independently of each other. Where multiple pairs of such loci showed synteny between separate linkage groups, we inferred these regions of the genome to be broadly homeologous.

Finally, we conducted a BLAST^46^ search of all tag sequences against the full genome sequences of barley, *Brachypodium* and rice, to determine whether syntenic chromosome regions could be identified. Matches were restricted to e < 10^−15^, and any tag with more than ten matches was eliminated.

## Results

### Map construction

**Figure 1:**
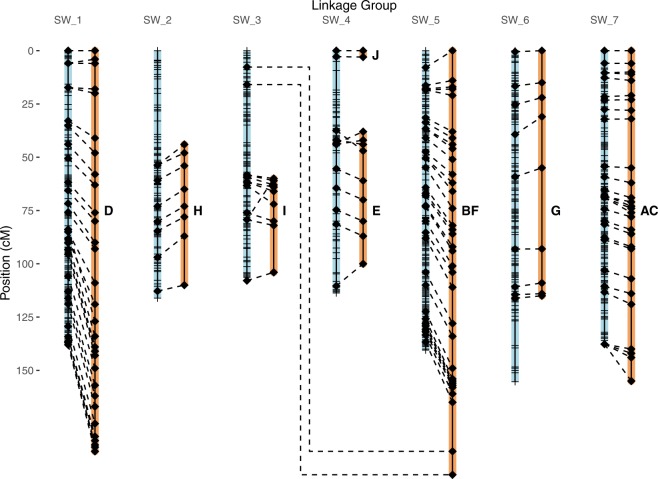
GBS linkage map of diploid *A*. *strigosa* (C.I. 3815) x *A*. *wiestii* (C.I. 1994) RIL population (blue, left), aligned to the RFLP map (orange, right) of Portyanko *et al*.^29^ from the same population. Linkage distances shown here were calculated after removal of likely GBS genotyping errors. Diamonds represent positions of common RFLP markers, while hatches indicated bins of other GBS markers.

We identified 6651 segregating loci in the SW mapping population, of which 3170 passed stringent filtering for use in map construction (Supplementary Table S1). MSTMap returned seven linkage groups at *e* = 10^−10^, corresponding to the expected seven chromosomes. Initial map length was estimated at 1850 cM, but following removal of 2285 putative errors (0.75% error rate), this was reduced to 905 cM (Fig. 1). The corrected markers clustered into 754 unique bins with an average inter-marker distance of 1.2 cM, a maximum gap of 11.5 cM, and only 15 gaps of >5 cM. All but five of the 204 RFLP markers from the original map^29^ were placed on the new GBS map, and these placements aligned with the original linkage groups (Fig. 1).

**Figure 2:**
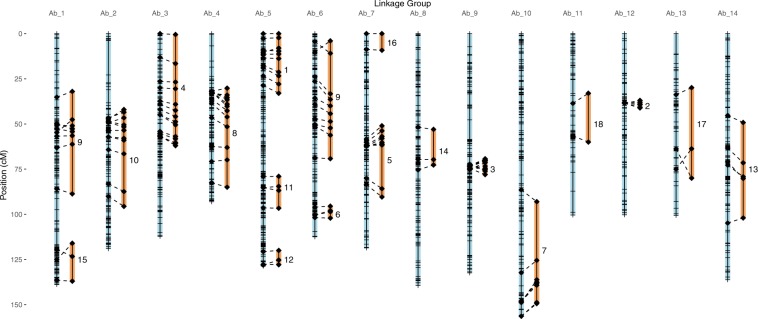
GBS linkage map (blue, left) of tetraploid *A*. *barbata* mapping population aligned to the AFLP map (orange, right) of Gardner and Latta^39^. Note that several linkage groups of the fragmentary AFLP map align to the same GBS chromosome. This map is derived from the double restriction digest (PstI-MspI) GbS library, with linkage distances calculated after removal of likely GBS genotyping errors. Diamonds represent position of AFLP markers, while hatches indicated bins of other GBS markers.

In *A*. *barbata*, the Haplotag pipeline identified 9148 segregating loci from the double-digest GBS library, of which 4015 passed stringent filtering for use in map construction. MSTMap returned 14 linkage groups for *A*. *barbata* at a threshold of *e* = 10^−12^. The initial map length of roughly 2500 cM was reduced to 1688 cM after the removal of 12,789 genotyping errors (error rate 1.8%). The 4015 markers clustered into 1529 bins, with an average inter-marker distance of 1.1 cM, but with somewhat more gaps than the SW map above. There is a gap of 20 cM on SW_2, and some 49 gaps of >5 cM (Fig. 2). All of the 129 AFLP markers of Gardner and Latta^39^ could be placed on the newer GBS map, allowing alignment of that partial map with the present map (Fig. 2).

The single restriction digest GBS library obtained fewer markers - only 3134 segregating loci, of which 1730 passed stringent filtering. These markers had greater read depth (median 29/locus as opposed to 11/locus in the two-enzyme library). However, there was very little overlap in the loci revealed by the two library preps – only 369 biallelic marker loci were identified in both libraries (Supplementary Table S2). Stringently filtered loci from the single restriction digest library gave an independently constructed *A*. *barbata* linkage map, which also contained 14 linkage groups and which closely aligned with the double digest GBS map (Supplementary Fig. S1). Since the two independently derived maps are thus concordant, markers from the single-enzyme library were placed on the double enzyme map for all further comparisons

We also identified 8700 segregating PAVs in SW, and over 30,000 segregating PAVs in *A*. *barbata*. The vast majority of the secondary (less stringently filtered) biallelic loci and most of the PAVs could be placed on the maps at <10% recombination with the closest markers (Supplementary Methods). Compared with biallelic loci, however, the PAVs showed greater recombination with the mapped markers, consistent with the greater error in determining PAV genotypes. Nevertheless, with all loci included, more than 40,000 markers were mapped in *A*. *barbata*, and nearly 15,000 in SW.

### Homologs, paralogs and homeologs

**Figure 3:**
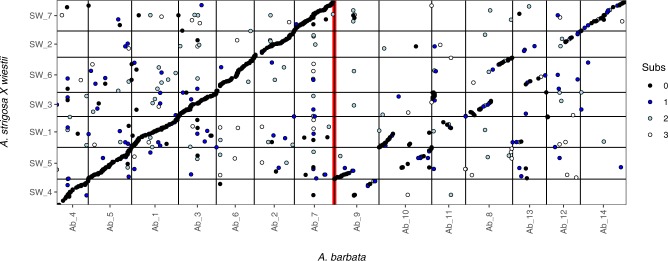
Dot-plot showing alignment of linkage groups between diploid *A*. *strigosa* (C.I. 3815) × *A*. *wiestii* (C.I. 1994) RIL population and tetraploid *A*. *barbata* linkage maps. Dots are coloured to indicate the number of base substitutions (black = perfect match; blue, grey, and white represent 1, 2, or 3 mismatches, respectively) between matching Tag sequences. Linkage groups have been re-ordered to emphasize the pattern.

We identified a total of 3732 homologous tag pairs from 3100 loci across the SW and *A*. *barbata* mapping populations (Table 1). These homologous markers mapped widely across all seven of the SW linkage groups, but in *A*. *barbata*, most of these homologs mapped to only seven of the linkage groups (Fig. 3). Moreover, these homologies were syntenic across linkage groups, indicating a direct 1:1 correspondence between the seven chromosomes of the A-genome diploids, and half of the 14 chromosomes in the AB tetraploid *A*. *barbata*. We have therefore designated these seven linkage groups as the A-genome of *A*. *barbata*, and assigned them corresponding numbers, 1 through 7. (Fig. 3.) The remaining seven linkage groups (Ab_8-14) in *A*. *barbata* thus presumably represent the B genome. These share many fewer homologous tags, giving a lower Dice similarity, with the SW linkage groups, and a larger fraction of those that are shared have base pair mismatches (Table 1). However, those that are shared are consistent with the pattern of homeology within the *A*. *barbata* genome (below).

**Table 1:** Number of putatively homologous tag pairs between subgenomes within linkage maps of diploid, tetraploid and hexaploid oats.

	*A*. *strigosa* x *A*. *wiestii* (2n = 2 × = 14)	*A*. *barbata* (2n = 4 × = 28)		Totals^a^
	A genome	A genome	B genome	
*A*. *strigosa* × *A*. *wiestii*				
A genome	NA (NA)	2420/815 (0.1114)	155/342 (0.0251)	22018
*A*. *sativa* (2n = 6× = 42)				
A genome	417/337 (0.0396)	619/494 (0.0427)	79/173 (0.0149)	16042
D genome	188/270 (0.0215)	222/432 (0.0231)	90/197 (0.0150)	20572
C genome	10/75 (0.0042)	22/117 (0.0051)	9/45 (0.0030)	18680
Totals^a^	22018	36075	17742	

The number of putative homologies is indicated separately for exact sequence matches, and for pairs showing 1–3 bp mismatches. Dice similarities, which account for the different number of tags in each genome are given in brackets.

^a^Total number of markers from each subgenome. Thus, for example, out of a total of 22018 mapped tags in the *A*. *strigosa* x *A*. *wiestii* mapping population, and 36075 tags mapped to the A genome of *A*. *barbata*, 2420 identical tags and 815 pairs showing 1–3 base mismatches were observed. This gives a total of 3235 putatively homologous tags for a Dice similarity of 0.1114.

**Figure 4:**
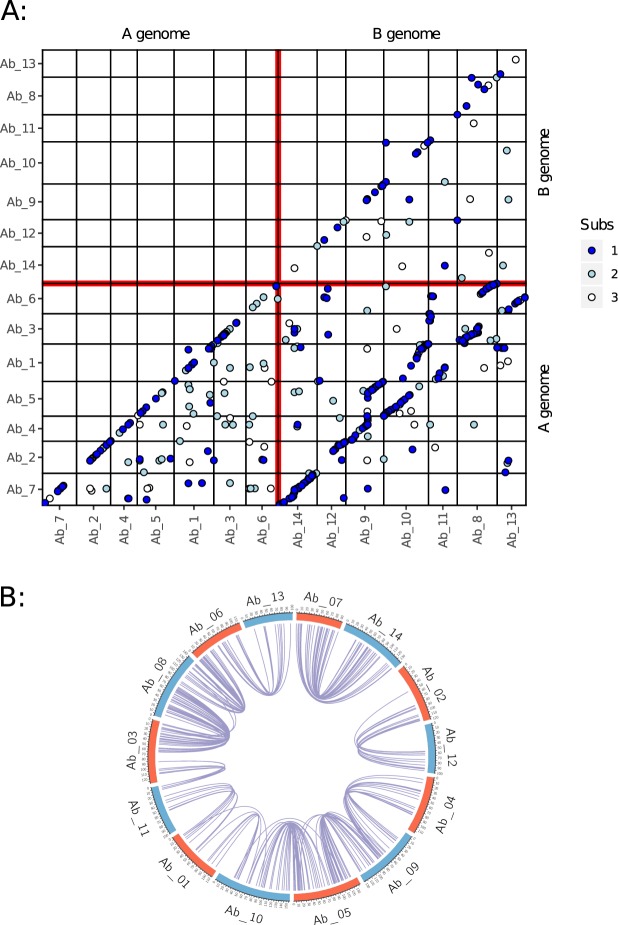
Paralogs and homeologs within the *A*. *barbata* genome. (**A**) Dot-plot of all paralogous loci detected with GBS. Linkage groups have been re-ordered to emphasize the pattern, and colour represents the number of base pair substitutions between the tag sequences of paralogous loci (blue, grey, and white represent 1, 2, or 3 mismatches, respectively). (**B**) Circos^58^ plot highlighting the pattern among linkage groups having more paralogous markers than expected by chance. A and B genome assignments from Fig. 3 are coloured red and blue respectively. Paralogous loci are joined by blue curved lines.

Within the *A*. *barbata* genome, there are 644 multi-locus tag clusters (in which two or more separate segregating loci or PAVs can be identified) which represent putative paralogs or homeologs. A randomization test (Supplementary Table S3) indicated that these pairs are not randomly distributed across the genome. First, locus pairs that map to the same linkage group are significantly over-represented. These typically map very close to each other (frequently in the same map bin, and rarely more than 2 cM apart - Fig. 4a) and are likely paralogs that resulted from small tandem repeats. More interestingly, several pairings of one linkage group from the A genome (as identified above), and one from the B-genome, have more paralogs than expected by chance alone. In these linkage groups, the paralogs show syntenic arrangements on the two linkage groups indicating large regions of homeology between the A and B genomes (Fig. 4a). In one instance, Ab_7 and Ab_14 show syntenic arrangement along the length of both linkage groups. However, most B genome chromosomes show substantial evidence of translocation when compared to A genome chromosomes. For example, Ab_4 is syntenic with one arm of Ab_9, the other arm of which is syntenic with Ab_5. This pattern continues through most of the *A*. *barbata* linkage groups (Fig. 4b) with alternating homeologies between the A and B genomes: 4-9-5-10-1-etc. The weak homology between the B-genome of *A*. *barbata* and the diploid A genome (Fig. 3) is largely consistent with this pattern of homeology within *A*. *barbata*. For example, Ab_14 shows fewer homologies to SW_7 than does its A genome homeolog Ab_7 (and fewer of these are identical sequence matches – Table 1), but both of these homeologous linkage groups are syntenic with the same linkage group in SW.

### Comparison to *A. sativa*

**Figure 5:**
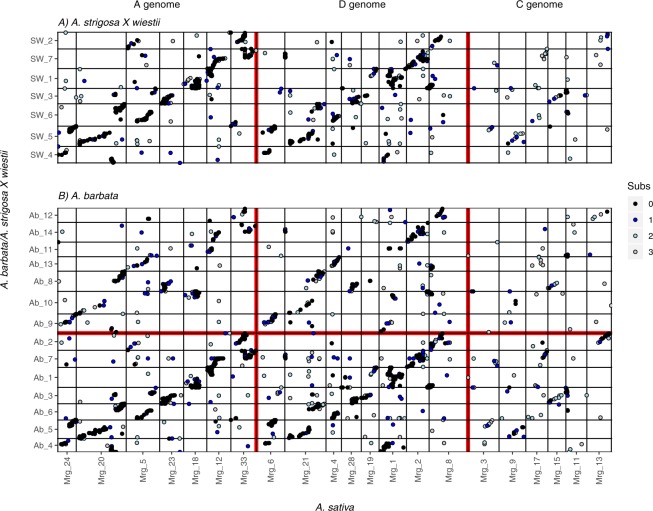
Dot-plots showing alignment of the consensus linkage map from *A*. *sativa*^20,47^ with the diploid *A*. *strigosa* × *A*. *wiesteii* map and with the tetraploid *A*. *barbata* map. Linkage groups have been re-ordered to emphasize the pattern, and colour represents the number of base pair substitutions between the tag sequences of homologous loci (black = perfect match; blue, grey, and white represent 1, 2, or 3 mismatches, respectively). Genome assignments of linkage groups in *A*. *sativa* following Yan *et al*.^21^ are shown across the top, with linkage group assignments at the bottom. Groups Mrg18 and Mrg19 are shown as A and D genomes, respectively, although they contain large translocations from the hexaploid C genome^21^.

Given the strong synteny between SW and the A genome of *A*. *barbata*, it is not surprising that they both showed similar patterns of homology with *A*. *sativa* (Fig. 5). The largest number of homologies occurs between the A genomes of SW/*barbata* and the A genome of *A*. *sativa*, with less similarity to D and least to the C-genome (Table 1). A similar pattern occurs between the B genome and the subgenomes of *A*. *sativa*, but with less homology than for the SW/*barbata* A genome.

**Figure 6:**
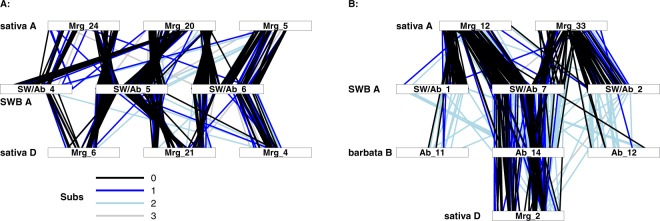
Example alignments of linkage groups from *A*. *strigosa*, *A*. *wiestii* and *A*. *barbata* against the A and D genomes of *A*. *sativa*. (**A**) alignment of LGs 4, 5, and 6 from the *A*. *sativa* × *A*. *wiestei* and *A*. *barbata* maps (labelled “SWB”) against the corresponding A and D genome LGs of *A*. *sativa*. Note that Mrg24 and Mrg6 are homeologs^20^ as are Mrg20 and Mrg21 as well as Mrg4 and Mrg5. (**B**) alignment of LG7 (A genome of SW and *A*. *barbata*), LG 14 (B genome of *A*. *barbata*), and Mrg2 (D genome of *A*. *sativa*) with Mrg12 and 33 both of the A genome of *A*. *sativa*. Linkage groups have been standardized to unit length, and line colour indicates the number of base pair differences between homologous tag sequences.

However, only a few linkage groups in *A*. *sativa* show synteny along their entire length with SW/*barbata* A-genome linkage groups (Fig. 5). For example, Mrg2 shows synteny to SW_7 and Ab_7, but most of the hexaploid linkage groups show apparent translocations, some of which are remarkably consistent between the A and D genomes of *A*. *sativa*. For example, alternate ends of linkage groups 4, 5 and 6 in SW/*barbata* appear to be rearranged as parts of Mrg24, 20 and 5 in the A genome of *A*. *sativa*, and as parts of Mrg 6, 21, and 4 in the D genome of *A*. *sativa* (Fig. 6A). Notably, these same pairs of hexaploid groups (Mrg24/6, 20/21, and 5/4) were identified as A/D genome homeologs by Chaffin *et al*.^20^. Overall, the D and A genomes of *A*. *sativa* show a parallel set of rearrangements compared to the SW/*barbata* genomes (Fig. 5).

Similarly, the B genome of *A*. *barbata* shows homology to the *A*. *sativa* genome that parallels that of the SW/*barbata* A genome (Fig. 5). For example, *A*. *barbata* Ab_14 shows the same homology with *A*. *sativa* Mrg2 (D genome) as does its A genome homeolog, SW_7/Ab_7 (Fig. 6B). This synteny appears to have been rearranged in the A genome of *A*. *sativa* between Mrg12 and Mrg33 (Fig. 6B).

Only a faint signal of homology to the C genome is observed (Fig. 5), in that few pairs of linkage groups show more homologous tags than expected by chance (for example, SW_3/Ab_3 with *A*. *sativa* Mrg15). We note that as the number of putative homologs declines, indicating more dissimilar genomes, the quality of the sequence match declines in parallel, as indicated by a greater proportion of those homologs containing base pair mismatches (Table 1).

**Figure 7:**
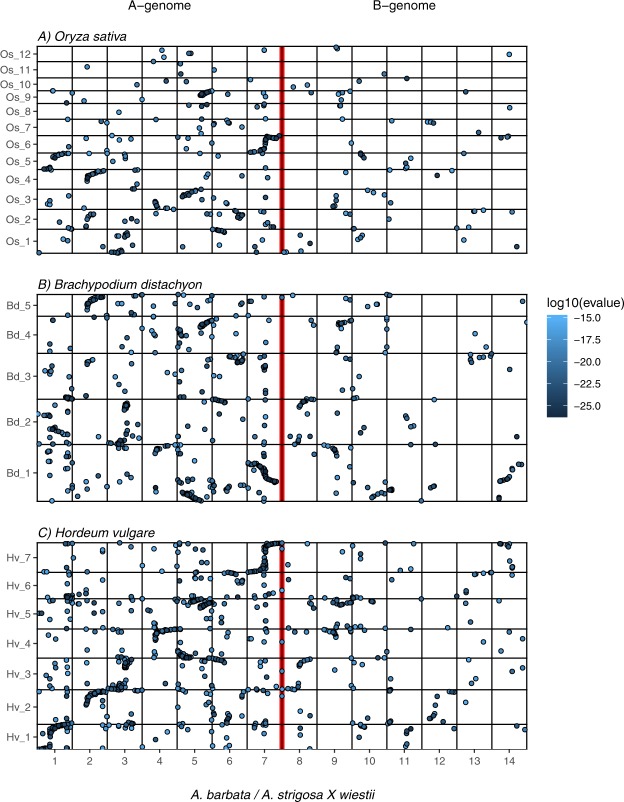
Dot-plot comparison of the *Avena barbata* and *A*. *strigosa* × *A*. *wiestii* (SW) linkage maps with the rice (top), *Brachypodium* (middle) and barley (bottom) genomes. LG’s 1–7 include markers from both *A*. *barbata* and SW with blast hits in the target genomes. X axis in recombination units (cM) with linkage groups standardized to unit length. Y axis in base pairs. Blast hits were filtered to max e-value of 10^−15^, and tags matching more than 10 locations in the target genomes were removed.

There is substantial synteny between barley chromosomes and several of the A genome linkage groups (Fig. 7). Although this has clearly been interrupted in some chromosomes, we have numbered the A genome linkage groups in SW and *A*. *barbata* to reflect this similarity with barley. Fewer areas of synteny are seen with the B-genome of *A*. *barbata*, in part because far fewer markers were identified in the B-genome. Some regions of synteny are seen for *Brachypodium* and rice, but since these are more distantly related to *Avena* than is barley, fewer regions of synteny are evident.

## Discussion

Our GBS data have permitted a considerable improvement in the maps of diploid and tetraploid *Avena* species. The greatly-increased density of these maps, together with the high numbers of secondary GBS loci and PAVs, will provide future opportunities for comparative mapping, and will allow integration of these maps with future genome sequencing efforts. Our GBS map (905 cM) allows some slight extension of SW_2, 3, 4 and 6, compared to the original RFLP map of *A. strigosa* X *wiestii*^29^ which covered some 862 cM. However, the improvement comes primarily in the much higher density of markers on the GBS map, since the RFLP map had numerous gaps of up to 40 cM.. A much greater improvement is seen in extending the tetraploid *A*. *barbata* map by some 2.5 fold compared to an earlier AFLP map, which Gardner and Latta^39^ acknowledged was incomplete.

We would expect that the tetraploid genome would be roughly twice the size of the diploid, and the *A*. *barbata* map does span slightly less than twice the length of the SW map. Polyploid *Avena* species have slightly less DNA than predicted from the composite diploid genomes^48^, an observation consistent with ‘genome downsizing’ in polyploids^3,49^. The relative sizes of the linkage maps (905 *vs*. 1688 cM) present a strkingly close match to the relative DNA contents of *A*. *strigosa* and *A*. *wiestii* (9.07 pg/2 C) versus *A*. *barbata* (16.45 pg/2 C). This would suggest that any genomic changes since polyploidization have not altered the overall frequency of crossovers per megabase of DNA.

**Figure 8:**
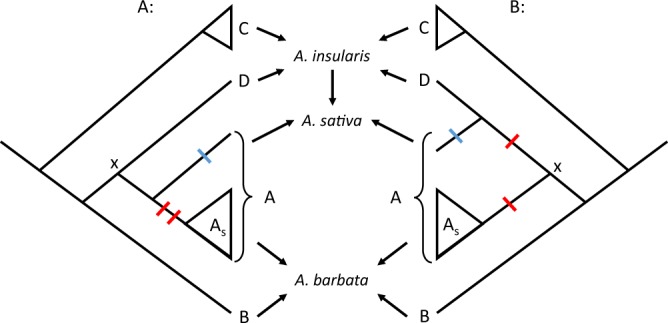
Two hypothetical relationships among the diploid ancestors of polyploid oats prior to the hybridization and polyploidization events (arrows) creating *A*. *barbata*, *A*. *insularis* and *A*. *sativa*. Left: The A genome diploid ancestor of *A*. *sativa* is more closely related to A_s_ diploids including *A*. *strigosa* and *A*. *wiestii*. Under this view, the ancestor at the node labelled “x” likely exhibited the genome arrangement seen in Mrg4/5, Mrg20/21, Mrg6/24, and a series of translocations (red hatches) in the common ancestor of A_s_ diploids led to the arrangement in SW_4–6 (Fig. 6a). Right: The A and D genome diploid ancestors of *A*. *sativa* are each other’s closest relatives. If this hypothesis is correct, then the contrasting arrangements in Mrg4/5, Mrg20/21, Mrg6/24 and SW_4–6 represent the accumulation of translocations (red hatches) as each lineage diverged from ancestor x. In either case, the unique arrangement of Mrg12 and Mrg33 in the A genome of *A*. *sativa* occurred (blue hatch) after that diploid ancestor diverged from the D genome ancestor.

The striking homology and synteny between the SW linkage map and Ab_1–7 of *A*. *barbata* strongly suggests that the A genome of *A*. *barbata* is closely related to the A genome diploids *A. strigosa* and *A*. *wiestii*. Seven *Avena* species share the A_s_ designation, and chloroplast phylogenies^23,24,26^ place *A*. *barbata* within this clade, indicating that an A_s_ genome diploid was the maternal ancestor of *A*. *barbata*. Multi-dimensional scaling of GBS markers^21^ also suggested a closer similarity of *A*. *barbata* to *A*. *hirtula* and *A*. *atlantica* than to *A*. *strigosa* or *A*. *wiestii*, and *A*. *hirtula* has been suggested as the A genome ancestor of *A*. *barbata*^50^. The close similarity between Ab_1–7 and SW_1–7 imply a recent divergence between *A*. *barbata’s* A genome and its diploid relatives (Fig. 8).

The greatly reduced number of shared loci between the *A*. *barbata* B genome and the genome of SW (Fig. 3, Table 1) suggests a much more distant relationship. Moreover, extensive chromosomal rearrangements distinguish the A and B genomes of *A*. *barbata*, as evidenced by the fact that few pairs of linkage groups are syntenic along their entire length (Fig. 4). These findings imply a much earlier divergence of the B genome from the main A genome lineage (Fig. 8) Thus, our results make clear that *A*. *barbata* is an allopolyploid that arose from the hybridization of two species with distinct chromosome arrangements, rather than an autopolyploid, resulting from genome doubling within a single species.

Yet if the divergence between the A genome of *A*. *barbata* and its diploid ancestor was relatively recent, then the hybridization and polyploidy event that formed *A*. *barbata* must also be recent. While no B-genome diploid oat species has been identified, it must have been extant up until the time of this hybridization, though it may have been quite rare. We also note that the lower density of markers on the B genome chromosomes suggests the possibility of less polymorphism in loci on the B genome. This in turn suggests that the B genome diploid ancestor may have been a genetically homogeneous species that hybridized multiple times^51^ with a more common and genetically heterogeneous A genome species.

We find no evidence that exchanges between A (Ab_1–7) and B (Ab_8–14) genomes have occurred since the formation of polyploid *A*. *barbata*. Had they occurred, such exchanges would have created circumstances where different parts of a single linkage group in SW would show strongest homology to A *vs*. B genome linkage groups in *A*. *barbata*. No such case is seen in Fig. 3, although we recognize that small gene conversion events may fall below the resolution of our maps. By contrast, such inter-genomic exchanges are typical of hexaploid oat^20,21^ and other allopolyploids^4,7,52^. The lack of intergenomic translocations implies that the structural differences between the A and B genomes must have been present in their diploid ancestors prior to hybridization and polyploidization. Structural rearrangement of diploids has been documented in other grass species. Notably, rye (*Secale cereale*) exhibits several translocation events derived from the putative Triticeae ancestral karyotype^14^, and the diploid wheat relative *Aegilops markgrafii* exhibits rearrangements compared to the D genome of wheat^15^. Intriguingly, in both of these examples, phylogenetic analysis suggests homoploid hybridization in the ancestry of the rearranged diploids.

There is a striking pattern to the homeologies between the A and B genomes that might be called an ‘enchainment of translocations’ (Fig. 4b). This pattern has a close parallel in the A and C genomes of tetraploid *Brassica napus*^12^. In that system, the contrasting genome arrangements are present in the diploid ancestors, *B*. *rapa* and *B*. *oleracea*. Similarly, we contend that the different arrangements in the genomes of polyploid oats were present in the diploid ancestors, rather than being a product of polyploidy. Rearrangements in the genomes of *Brassica* diploids have been traced to an earlier round of polyploidization and re-diploidization some 5–20 mya^12,53,54^. However, there is currently no evidence suggesting paleopolyploidy in the lineage leading to modern oats (at least none more recent than the ancestral grass karyotype)^55^, though admittedly the genomic resources to detect such a paleopolyploidy event in *Avena* are not fully developed. Alternatively, we can speculate that a series of reciprocal translocations similar to the 4 AL-5AL-7BS cyclical translocation in wheat^56^ may have structured the *A*. *barbata* genome. In wheat an early 4 A/5A reciprocal translocation was followed by a subsequent 4A/7B exchange. These are non-homeologous exchanges, the first of which occurred in the diploid ancestor, *Triticum urartu*. The pattern in *A*. *barbata* appears to be longer, and not fully cyclical – a pattern which could arise if one of the translocations occurred between highly asymmetrical chromosomal blocks (sometimes termed “non-reciprocal”, though see Schubert and Lysak^57^). As in wheat, this pattern presumably formed from a succession of pairwise translocations, not all of which necessarily took place in the B genome. Rather, as the A and B genomes diverged from a common ancestral arrangement, translocations in both lineages likely led to the present pattern of homeology. Supplementary Fig. S2 presents one possible sequence for these translocations.

While the A genomes of SW and *A*. *barbata* are very similar, the A-genome of *A*. *sativa* is much less similar to either (Fig. 5, Table 1), consistent with suggestions that it is derived from a different sub-lineage within the A genome diploids, such as the A_l_ genome of *A*. *longiglumis*^21^. Moreover, this A genome diploid ancestor of *A*. *sativa* likely diverged from the A_s_ genome diploids much earlier than the A genome diploid ancestor of *A*. *barbata* (See Fig. 8) Indeed, the D genome of *A*. *sativa* is only slightly less similar to SW and *A*. *barbata* than is the *A*. *sativa* A genome. The B-genome of *A*. *barbata* shows still fewer homologies to the A genome of SW (Table 1), suggesting that it is more distantly related (Fig. 8). It is plausible that the B and D genomes are derived from other sub-types of A-genome diploids.  Of course, the C genome is least similar to any of the other genomes, which is consistent with phylogenetic analyses^23,24,26^ that consistently place the C-genome diploids as a separate clade (along with CC autotetraploid *A*. *macrostachya*).

We have inferred a recent hybrid origin of *A*. *barbata* because of the recent divergence between its A genome diploid ancestor and the other A_s_ genome diploids. The lack of intergenomic translocations in *A*. *barbata* (Fig. 3) is consistent with such a recent origin. By the same reasoning, an earlier divergence of *A sativa*’s A genome diploid ancestor implies that the hybridization event that formed *A*. *sativa* may be older than the one that formed *A*. *barbata*. An earlier formation of *A*. *sativa* than *A*. *barbata* may explain why intergenomic translocations are seen frequently in *A*. *sativa*^20,21^ but not *A*. *barbata*, simply because there has been less time for such exchanges to occur in *A*. *barbata*. In *A*. *sativa*, more translocations are seen between the C and D genomes than between either subgenome and A^20,21^. This is consistent with the fact that the CD tetraploid ancestor of *A*. *sativa* (likely *A*. *insularis*)^21^ must have evolved prior to the time *A*. *sativa* arose, giving the C and D genomes more time to undergo inter-genomic translocations.

Comparisons with *A*. *sativa* (Fig. 5) reveal similar patterns of enchained translocation to those seen in *A*. *barbata*, although these do not seem to extend across the *A*. *sativa* genome to the same extent as *A*. *barbata*. This may be in part because ongoing translocations between the homeologous genomes in the hexaploid^20^ have obscured the similarities of pattern within genomes. Remarkably, the A and D genomes of *A*. *sativa* often show the *same* rearrangements relative to the A genome of *A*. *strigosa*, *A*. *wiestii* and *A*. *barbata* (Figs 5, 6A). These appear to be parallel rearrangements within the A and D genomes, and thus are difficult to ascribe to intergenomic translocations following polyploidization. Instead, we find it more parsimonious to hypothesize that the distinct chromosome arrangement in the A and D genomes of *A*. *sativa* was present in the common ancestor of the diploid lineages that gave rise to those genomes (labelled “x” on Fig. 8). Since the A genome of *A*. *sativa* shows more homologies with the A genome of SW/*barbata* than does the D genome (Table 1), this suggests that the D genome diverged prior to the divergence of the A genome of *A*. *sativa* and SW/*barbata* (Fig. 8A). Under this view, the arrangement seen in the A genome of SW and *A*. *barbata* is derived from that of the *A*. *sativa* A genome ancestor, through successive translocations in SW_4,5,6. This hypothesis implies that a series of translocations took place along a relatively short branch of the phylogeny. Alternatively, if the common arrangement of the A and D genomes in *A*. *sativa* is more indicative of common ancestry than their Dice similarities to SW, then the diploid A and D genome ancestors of *A*. *sativa* may be more closely related to each other than either is to the SW/*barbata* A genome ancestor (Fig. 8B). In this case, the different arrangements in SW/*barbata* vs the A and D genomes of *A*. *sativa* would be the cumulative result of translocations along both of the lineages as they diverged from some unknown ancestral arrangement at node x (Fig. 8B).

It would of course, be valuable here to identify an ancestral genome arrangement in the common ancestor of the A, B and D genomes. This is beyond the scope of this study, but one suggestion is tempting. Linkage groups SW_7 and Ab_7 (A genome), Ab_14 (B genome) and Mrg2 (D genome) all show strong homology and are syntenic along their entire length. Indeed, we note that SW_7 and Ab_7 show strong collinearity with barley chromosome 7 (and extends even to *Brachypodium* Bd_1 and rice Os_6 – Fig. 7). This strongly suggests that this chromosome has an ancestral arrangement. By contrast, Mrg12 and Mrg33 from the A genome of *A*. *sativa* exhibit a unique rearrangement of this chromosome (Fig. 6B). Parsimony thus suggests that this rearrangement is autapomorphic to the A genome ancestor of *A*. *sativa* prior to hybridizing with *A*. *insularis* (Fig. 8). Testing this suggestion, and fully inferring the arrangement of the ancestral genome would likely require extensive sequence data across multiple ploidy levels.

What our maps do reveal is substantial karyotypic variation among oats. This diversity has frustrated efforts to ascribe a consistent chromosome nomenclature across the genus. We have been able to apply a consistent nomenclature to SW and half of the *A*. *barbata* linkage groups, but there is no 1:1 correspondence to the B genome, or to the subgenomes of *A*. *sativa*. Not only have we found evidence that the B genome is indeed a distinct entity with a distinct chromosome arrangement, we also find evidence that the A genome in *A*. *sativa* has a further different karyotype from the A_s_ diploid genome of SW and *A*. *barbata*. This diversity is most parsimoniously explained if we hypothesize that rearrangements took place within the diploid ancestors of oats prior to the formation of allopolyploids. In this view, these rearrangements do not result as a consequence of polyploidy. Since new sequencing technologies may soon permit routine *de novo* analysis of complete genomes, we suggest that the largely-unknown structure of most of the 30+ *Avena* species will provide a rich arena to test hypotheses regarding karyotype evolution across a polyploid series.

### Supplementary information

Dataset 1

Supplementary Info

## Data Availability

GBS raw sequence read data are deposited at the NCBI short read archive under project numbers PRJNA517481 for the diploid the *A*. *strigosa* X *wiestii* mapping population, and PRJNA517323 for *A*. *barbata*. Genotype calls for each recombinant inbred line are included in Supplementary Data.
