# Supplementary material for: Comparative linkage mapping of diploid, tetraploid, and hexaploid Avena species suggests extensive chromosome rearrangement in ancestral diploids
Source: Sci Rep. 2019 Aug 23;9:12298. doi: 10.1038/s41598-019-48639-7 (PMC6707241; doi:10.1038/s41598-019-48639-7)

Supplementary Materials

Comparative linkage mapping of diploid, tetraploid, and hexaploid *Avena* species suggests extensive chromosome rearrangement in ancestral diploids.

Robert G. Latta[1*]
Wubishet A. Bekele[2]
Charlene P. Wight[2]
Nicholas A. Tinker[2]

1 Dept. of Biology, Dalhousie University, 1355 Oxford St., Halifax, NS, B3H 4R2, Canada. Phone: 902-494-2737, Fax: 902-494-3736, Email: Robert.Latta@Dal.ca.
2 Ottawa Research and Development Centre, Agriculture & Agri-Food Canada, 960 Carling Ave., Ottawa, Ontario, K1A 0C6, Canada.

* Author for Correspondence.

Contents:

Description of Supplementary Data (Accompanying .xlsx file)
- Genotype calls for all loci in both mapping populations
- Error calls
- Paralogs and homologs

Supplementary Methods
- MSTMap
- Haplotag Clusters, Paralogs and Presence/Absence Variants (PAVs)
- Mapping Secondary Loci
- Error Filtering.
- Supp. Methods Figures A-D

Supplementary Tables
- Table S1. Number of Loci Detected
- Table S2. Overlap of single and double digest GBS libraries in *A. barbata*
- Table S3. Statistical significance of homologs/paralogs between pairs of linkage groups

Supplementary Figures
- Figure S1. Comparison of Single and Double digest GBS linkage map in *A. barbata*.
- Figure S2. Hypothesized evolutionary transition between A and B genome karyotypes

Description of Supplementary Data (Accompanying .xlsx file)

GBS raw sequence read data are deposited at the NCBI short read archive under project numbers PRJNA517481 for the diploid the *A. strigosa* X *wiestii* mapping population, and PRJNA517323 for *A. barbata*.

However we also want to make available the genotype calls, error calls, and homologs identified in this study, since these are likely to be more accessible to researchers wishing to extend/examine our work.

<u>1) GBS Markers mapped in *Avena barbata*</u>
For each biallelic locus and Presence-Absence Variant, we give the sequence of the GBS tags (alleles), the inferred position on the linkage map, and the genotype calls for all RILs.

<u>2) GBS Markers mapped in *Avena strigosa* X *wiestii*</u>
As above for the diploid mapping population

<u>3-5) Genotyping Error Calls</u>
For each of the stringently filtered loci that we used to construct the framework map, we screened the map for likely genotyping errors as described in Supplementary Methods. The data file lists the genotype calls for each RIL before and after error checking, and compares the inferred map positions for the corrected vs uncorrected linkage maps.

<u>6) Homologous Markers across maps</u>
Sorted by species, with the lower ploidy mapping population on the left. Gives name, and map location for each of the inferred homologous tag pairs, and the number of base pair mismatches

<u>7) Paralogs within *A. barbata*</u>
As above for Homologs between species. Sorted by map position with the lower number linkage group (e.g. Ab_1 vs Ab_12) on the left

Supplementary Methods

We present here some illustrations and details of our analysis. Note that figures referred to in these supplementary methods are labelled A-D, to distinguish them from figures S1, S2, *etc*. cited in the main text.

MSTMap

The first step of the MSTMap algorithm separates loci into linkage groups (prior to ordering the markers) based upon the number of recombination events observed between them. Since loci on separate chromosomes may show less than 50% recombination by chance, a threshold number of observed recombinations is set based on the probability of observing a given number of recombinations between unlinked loci. Then linkage groups are formed such that loci within a group have observed recombination less than the threshold to at least one other member of the group. Threshold is set by the researcher through a parameter, *e*, which defines the probability of observing a given recombination level by chance between unlinked markers (through a calculation given in Wu et al., 2014 [45]). A high value of *e* gives a single linkage group with numerous gaps of ~50 cM between adjacent markers. We gradually reduced *e* in successive runs until the correct number of linkage groups was obtained. Further reductions of *e* would eventually begin to break up linkage groups at some of the longer gaps between markers (such as the 20cM gap on Ab_2).

Haplotag Clusters, Paralogs and Presence/Absence Variants (PAVs)

The Haplotag pipeline (Tinker et al, 2016 [44]) treats the entire GBS tag sequence as a haplotype which may represent a segregating allele at a locus. This is in contrast to the SNP-based approach which considers each SNP as a separate (though of course possibly tightly linked) locus. Figure A shows an example of Haplotag output for a cluster of seven tags with similar sequences but with more than one SNP. These haplotypes can be resolved into three segregating biallelic loci and one segregating presence absence variant. Loci 1 and 2 appear to be tandem repeats – they show very tight linkage disequilibrium and map to the same position (Fig B). Locus 3 is unlinked to loci 1 and 2. Haplotag identifies these loci by eliminating tag pairings that could not be alleles at a single locus. For example, a model that posits the tags 140113 and 140115 (mesic alleles at locus 2 and 3) are alleles at a single locus would be rejected because it would be missing from too many taxa as well as exceeding the threshold for heterozygosity. The remaining Tag 140117 shows fixed differences between the parents (it is only present in the xeric ecotype), and shows Mendelian segregation. It also shows strong association with the xeric allele at loci 1 and 2 in the recombinants and maps near to those loci (Fig B)

Mapping Secondary Loci

Figure B illustrates our method for placing secondary loci (less stringently filtered), PAV's, and RFLP and AFLP markers on the linkage map. The recombination fraction is computed for each marker with each bin of the framework map. Recombination fractions are roughly 0.5 for most bins, indicating no linkage. Map positions are assigned to the bin with the lowest recombination fraction.

The traces show the loci and PAV from the cluster of tags shown in Fig A. Loci 1 and 2 (red and yellow) and the PAV (green) map very close to each other. Loci 1 and 2 have identical traces, but the increased error in the PAV gives a slightly higher minimum RF. Locus 3 from that cluster maps to a different chromosome (light blue). Most loci showed close linkage (98% of rf <=0.1, Fig C) to one bin of the framework map and extensive recombination (rf ~ 0.5) to most other bins.

Error Filtering.

During our map construction, the stringently filtered loci were further screened for likely genotyping errors after an initial map was inferred. Fig D shows genotype data for selected RILs over a short region of LG 1 in the *A. strigosa* X *wiestii* map. The GBS markers between the solid black lines were originally inferred to cover 6.7 cM. However, each recombination event in this region produced double recombination around a single marker – an unlikely occurrence. Moreover, each of these putative double recombinants was found in heterozygous state, which is also highly unlikely in a selfing organism. When the original genotype calls (left) are thinned for errors (by removing any double recombinant involving fewer than 3 markers within less than 2 cM) there is seen to be no recombination events among these markers, placing them all within a single "bin".

Figure A: Example output of the Haplotag pipeline for a cluster of GBS tags that resolve into three segregating loci and one Presence-Absence Variant (PAV). A sample of genotype calls is given on the next page.

A_barbata_Map_Output GBS passport file for tag cluster: RGLP53537

Cluster Consensus: TGCAGKGCCTGGCRCGAGGCCGTCGACTCNTGCGGCCTCCTRCGGGCCGACMTKCTCYCGYTCT

TagID	Count*	TGCAGKGCCTGGCRCGAGGCCGTCGACTCNTGCGGCCTCCTRCGGGCCGACMTKCTCYCGYTCT<br>1 2 3 4 5 6
140110	85	TGCAGGGCCTGGCACGAGGCCGTCGACTCCTGCGGCCTCCTACGGGCCGACC TGCTCCCGCTCT
140112	87	TGCAGGGCCTGGCGCGAGGCCGTCGACTCCTGCGGCCTCCTGCGGGCCGACA TTCTCCCGCTCT
140113	97	TGCAGGGCCTGGCGCGAGGCCGTCGACTCCTGCGGCCTCCTGCGGGCCGACC TGCTCCCGCTCT
140114	81	TGCAGGGCCTGGCGCGAGGCCGTCGACTCGTGCGGCCTCCTGCGGGCCGACC TGCTCCCGCTCT
140115	95	TGCAGGGCCTGGCGCGAGGCCGTCGACTCTTGCGGCCTCCTACGGGCCGACC TGCTCCCGCTCT
140117	85	TGCAGGGCCTGGCGCGAGGCCGTCGACTCTTGCGGCCTCCTACGGGCCGACC TGCTCTCGCTCT
140118	99	TGCAGTGCCTGGCGCGAGGCCGTCGACTCCTGCGGCCTCCTGCGGGCCGACA TTCTCCCGCTCT

*Count = number of taxa that contain this haplotype

(For details of model selection click HERE)

Best model #1 fits 183 genotypes, with 2% heterozygotes.

Consensus: TGCAGKGCCTGGCGCGAGGCCGTCGACTCCTGCGGCCTCCTGCGGGCCGACATTCTCCCGCTCT

Locus 1		TGCAGKGCCTGGCGCGAGGCCGTCGACTCCTGCGGCCTCCTGCGGGCCGACATTCTCCCGCTCT
TagID	Count	1 2 3 4 5 6
140112	87	TGCAGGGCCTGGCGCGAGGCCGTCGACTCCTGCGGCCTCCTGCGGGCCGACATTCTCCCGCTCT
140118	99	TGCAGTGCCTGGCGCGAGGCCGTCGACTCCTGCGGCCTCCTGCGGGCCGACATTCTCCCGCTCT

Best model #2 fits 179 genotypes, with 1% heterozygotes.

Consensus: TGCAGGGCCTGGCRCGAGGCCGTCGACTCYTGCGGCCTCCTACGGGCCGACCTGCTCCCGCTCT

Locus 2		TGCAGGGCCTGGCRCGAGGCCGTCGACTCYTGCGGCCTCCTACGGGCCGACCTGCTCCCGCTCT
TagID	Count	1 2 3 4 5 6
140110	85	TGCAGGGCCTGGCACGAGGCCGTCGACTCCTGCGGCCTCCTACGGGCCGACCTGCTCCCGCTCT
140115	95	TGCAGGGCCTGGCGCGAGGCCGTCGACTCTTGCGGCCTCCTACGGGCCGACCTGCTCCCGCTCT

Best model #3 fits 176 genotypes, with 1% heterozygotes.

Consensus: TGCAGGGCCTGGCGCGAGGCCGTCGACTCSTGCGGCCTCCTGCGGGCCGACCTGCTCCCGCTCT

Locus 3		TGCAGGGCCTGGCGCGAGGCCGTCGACTCSTGCGGCCTCCTGCGGGCCGACCTGCTCCCGCTCT
TagID	Count	1 2 3 4 5 6
140113	97	TGCAGGGCCTGGCGCGAGGCCGTCGACTCCTGCGGCCTCCTGCGGGCCGACCTGCTCCCGCTCT
140114	81	TGCAGGGCCTGGCGCGAGGCCGTCGACTCGTGCGGCCTCCTGCGGGCCGACCTGCTCCCGCTCT

….Cont'd

Fig A (cont'd)

Best selected models are on the left -------- haplotypes excluded from the selected locus model(s) are on the right

TaxaID	Project	TaxaName	Locus Model and haplotype IDs						
			Locus-1		Locus-2		Locus-3		No-model
			140112	140118	140110	140115	140113	140114	140117
M-2084	DeepRead	M-2084	0	30	0	28	34	0	0
M-1884	Parents	M-1884	0	11	0	9	8	0	0
M-286	Parents	M-286	0	5	0	10	7	0	0
M-786	Parents	M-786	0	8	0	4	8	0	0
X-189	DeepRead	X-189	55	0	23	0	0	26	22
X-187	Parents	X-187	10	0	6	0	0	7	9
X-287	Parents	X-287	9	0	6	0	0	0	5
X-589	Parents	X-589	5	0	2	0	0	4	6
F7-001	RILs	F7-001	4	0	7	0	8	0	3
F7-002	RILs	F7-002	6	0	9	0	0	4	10
F7-003	RILs	F7-003	0	9	0	8	12	0	0
F7-004	RILs	F7-004	7	0	5	0	6	0	2
F7-005	RILs	F7-005	10	0	5	0	3	2	8
F7-006	RILs	F7-006	6	0	11	0	0	2	8
F7-007	RILs	F7-007	0	8	0	12	0	15	0
F7-008	RILs	F7-008	8	0	6	0	10	0	5
F7-009	RILs	F7-009	0	6	0	4	3	0	0
F7-010	RILs	F7-010	0	13	0	4	12	0	0
F7-011	RILs	F7-011	0	2	0	0	2	0	0
F7-012	RILs	F7-012	7	0	7	0	5	0	5
F7-013	RILs	F7-013	11	0	3	0	9	0	4
F7-014	RILs	F7-014	7	0	3	0	13	0	7
F7-015	RILs	F7-015	0	11	0	10	0	6	0
F7-016	RILs	F7-016	0	5	0	5	7	0	0
F7-017	RILs	F7-017	0	12	0	7	15	0	0
F7-018	RILs	F7-018	0	13	0	9	0	11	0
F7-019	RILs	F7-019	7	0	2	0	3	0	4
F7-020	RILs	F7-020	0	5	0	4	5	0	0
F7-021	RILs	F7-021	0	4	0	2	4	0	0
F7-022	RILs	F7-022	12	0	7	0	0	2	10
F7-023	RILs	F7-023	0	12	0	6	6	0	0
F7-024	RILs	F7-024	0	7	0	5	12	0	0
F7-025	RILs	F7-025	0	8	0	9	22	0	0
F7-026	RILs	F7-026	0	14	0	12	0	8	0
F7-027	RILs	F7-027	7	0	11	0	0	5	9

Figure B. Traces of the recombination fraction for each Locus in Fig S1A, against each bin of the framework map

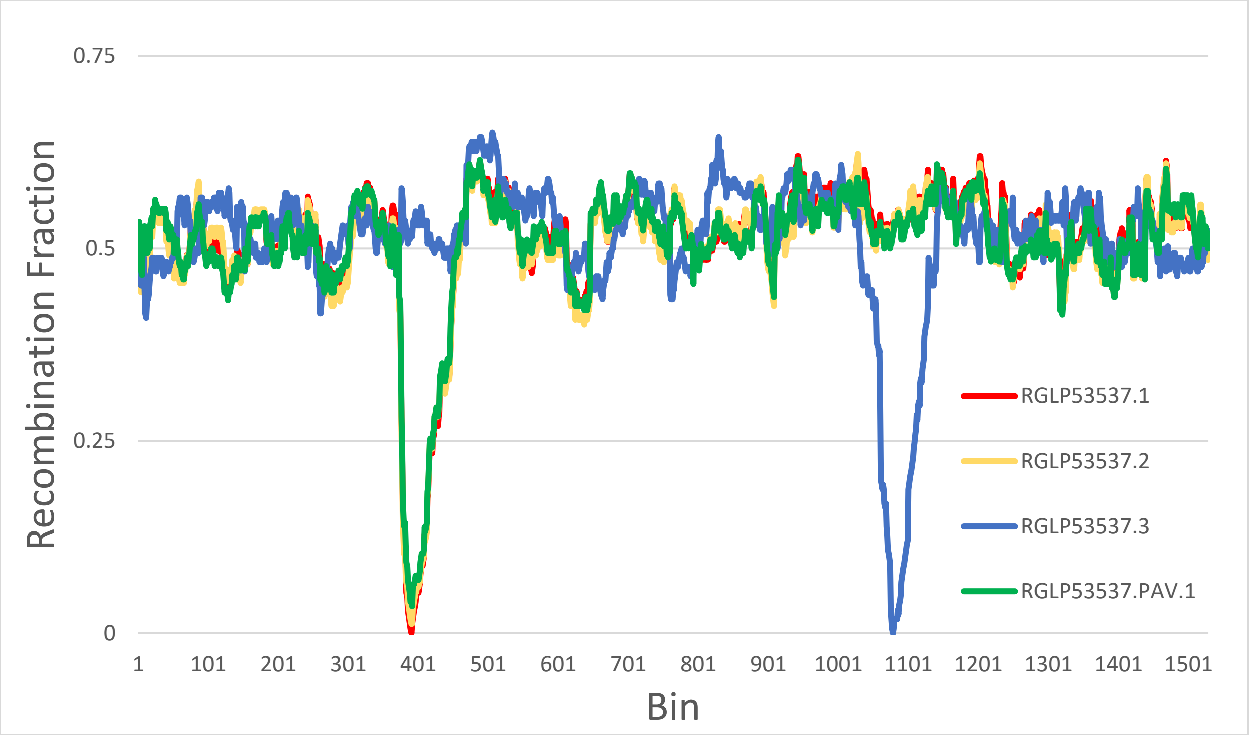

Fig C. Frequency distribution of recombination fraction between secondary loci and PAVs and the nearest marker of the framework map in *A. barbata* and *A. strgosa* X *wiestii*)

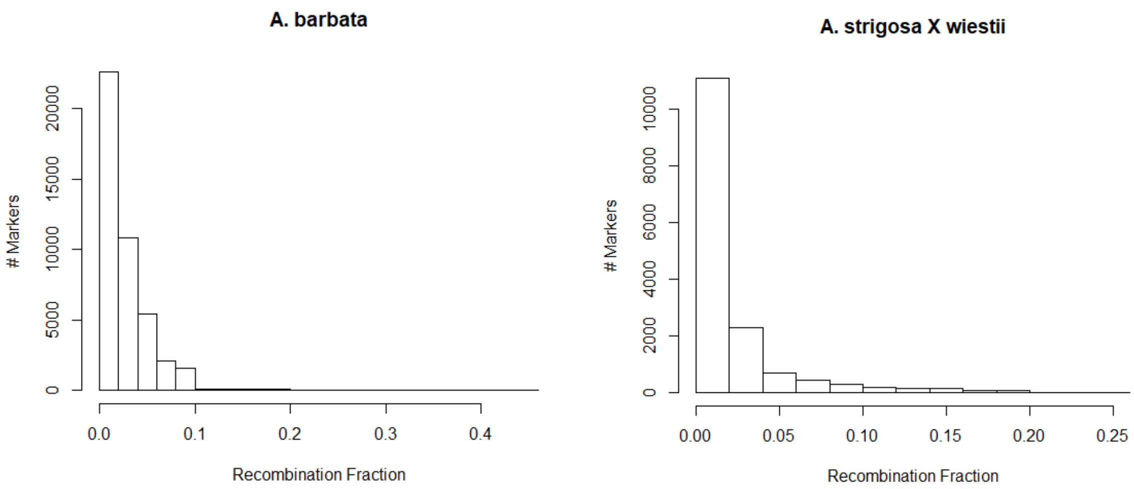

Figure D: Illustration of error checking in the framework map. A selection of RILs from the SW mapping population is illustrated. RILs have been selected to illustrate errors, thus RILs with errors are over-represented in this example. On the left of each pair is the original genotype call from Haplotag (1 = *wiestii* homozygote, 2 = heterozygote, 3- *strigosa* homozygote), while on the right are the genotype calls once errors have been removed and treated as missing data. The original map positions indicate 7 cM in the span indicated by the black lines, but this is mostly attributable to erroneous heterozygote calls inflating the rate of (double) recombination. The complete set of error calls are given in the Supplementary Data.

Locus	LG	Pos	NewPos	SW_002		SW_014		SW_015		SW_019		SW_024		SW_025		SW_031		SW_032		SW_036		SW_048		SW_069		SW_073		SW_078		SW_079		SW_080		SW_082		SW_091	
Oat19439.1	1	186	100.289	3	3	3	3	1	1	1	1	2	2	1	1	3	3	1	1	1	1	1	1	1	1	1	1	1	1	3	3	1	1	3	3	2	2
Oat1246.1	1	188.3	102.128	3	3	3	3	1	1	1	1	2	2	3	3	3	3	1	1	1	1	1	1	1	1	1	1	1	1	3	3	1	1	3	3	2	2
Oat3348.1	1	190.7	102.128	3	3	3	3	1	1	1	1	3	NA	3	3	2	NA	1	1	1	1	1	1	1	1	1	1	1	1	3	3	1	1	3	3	2	2
Oat27635.1	1	189.5	102.232	3	3	3	3	1	1	1	1	2	2	3	3	3	3	1	1	1	1	1	1	1	1	1	1	1	1	3	3	1	1	3	3	2	2
Oat13269.1	1	192	102.336	3	3	3	3	1	1	1	1	2	2	3	3	3	3	1	1	1	1	1	1	1	1	1	1	1	1	3	3	1	1	2	NA	2	2
Oat36708.1	1	193.7	103.088	3	3	3	3	1	1	1	1	2	2	3	3	3	3	1	1	1	1	1	1	1	1	1	1	1	1	3	3	1	1	3	3	3	3
Oat34471.1	1	194.3	103.088	3	3	3	3	1	1	1	1	2	2	3	3	3	3	1	1	1	1	1	1	1	1	1	1	1	1	3	3	2	NA	3	3	3	3
Oat29128.1	1	195.1	103.088	3	3	3	3	1	1	1	1	2	2	3	3	3	3	1	1	1	1	1	1	2	NA	1	1	2	NA	3	3	1	1	3	3	3	3
Oat3236.1	1	195.6	103.088	3	3	3	3	1	1	1	1	2	2	3	3	3	3	1	1	1	1	1	1	1	1	1	1	1	1	3	3	1	1	3	3	3	3
Oat4822.1	1	195.6	103.088	3	3	3	3	1	1	1	1	2	2	3	3	3	3	1	1	1	1	1	1	1	1	1	1	1	1	3	3	1	1	3	3	3	3
Oat13926.1	1	195.6	103.088	3	3	3	3	1	1	1	1	2	2	3	3	3	3	1	1	1	1	1	1	1	1	1	1	1	1	3	3	1	1	3	3	3	3
Oat36793.1	1	195.6	103.088	3	3	3	3	1	1	1	1	2	2	3	3	3	3	1	1	1	1	1	1	1	1	1	1	1	1	3	3	1	1	3	3	3	3
Oat37650.1	1	195.6	103.088	3	3	3	3	1	1	1	1	2	2	3	3	3	3	1	1	1	1	1	1	1	1	1	1	1	1	3	3	1	1	3	3	3	3
Oat40544.1	1	195.6	103.088	3	3	3	3	1	1	1	1	2	2	3	3	3	3	1	1	1	1	1	1	1	1	1	1	1	1	3	3	1	1	3	3	3	3
Oat19266.1	1	196.1	103.088	3	3	3	3	1	1	2	NA	2	2	3	3	2	NA	1	1	1	1	1	1	1	1	1	1	1	1	3	3	1	1	3	3	3	3
Oat18907.1	1	197.9	103.088	3	3	2	NA	1	1	1	1	2	2	3	3	3	3	1	1	1	1	1	1	1	1	1	1	1	1	2	NA	1	1	3	3	3	3
Oat28613.1	1	198.4	103.088	3	3	3	3	1	1	1	1	2	2	3	3	3	3	1	1	1	1	1	1	1	1	1	1	1	1	3	3	1	1	3	3	3	3
Oat4480.1	1	199.2	103.088	3	3	3	3	1	1	1	1	2	2	3	3	3	3	1	1	1	1	2	NA	1	1	1	1	1	1	3	3	1	1	3	3	3	3
Oat18906.1	1	200	103.088	3	3	3	3	1	1	1	1	2	2	3	3	3	3	2	NA	1	1	1	1	1	1	2	NA	1	1	3	3	1	1	3	3	3	3
Oat25536.1	1	201	103.626	3	3	3	3	1	1	1	1	2	2	3	3	3	3	1	1	3	3	1	1	1	1	2	NA	1	1	3	3	1	1	3	3	3	3
Oat1791.1	1	201.6	103.626	3	3	3	3	1	1	1	1	2	2	3	3	3	3	1	1	3	3	1	1	1	1	1	1	1	1	3	3	1	1	3	3	3	3
Oat35330.1	1	202.2	104.164	3	3	3	3	1	1	1	1	2	2	3	3	3	3	1	1	3	3	1	1	1	1	1	1	1	1	3	3	1	1	3	3	3	3
Oat37523.1	1	203.5	105.239	3	3	3	3	1	1	1	1	2	2	3	3	3	3	1	1	3	3	3	3	1	1	1	1	1	1	3	3	1	1	3	3	3	3
Oat2358.1	1	204	105.765	3	3	3	3	1	1	1	1	2	2	3	3	3	3	1	1	3	3	3	3	1	1	1	1	1	1	3	3	1	1	3	3	3	3
Oat4691.1	1	204	105.765	3	3	3	3	1	1	1	1	2	2	3	3	3	3	1	1	3	3	3	3	1	1	1	1	1	1	3	3	1	1	3	3	3	3
Oat6600.1	1	204	105.765	3	3	3	3	1	1	1	1	2	2	3	3	3	3	1	1	3	3	3	3	1	1	1	1	1	1	3	3	1	1	3	3	3	3
Oat15406.1	1	204	105.765	3	3	3	3	1	1	1	1	2	2	3	3	3	3	1	1	3	3	3	3	1	1	1	1	1	1	3	3	1	1	3	3	3	3
Oat20362.1	1	204	105.765	3	3	3	3	1	1	1	1	2	2	3	3	3	3	1	1	3	3	3	3	1	1	1	1	1	1	3	3	1	1	3	3	3	3
Oat34353.1	1	204	105.765	3	3	3	3	1	1	1	1	2	2	3	3	3	3	1	1	3	3	3	3	1	1	1	1	1	1	3	3	1	1	3	3	3	3

Supplementary Tables

Table S1. Number of biallelic and presence-absence variant (PAV) markers identified for each GBS library in each mapping population

		A. strigosa x *A. wiestii*	*A. barbata*	
		Double Digest	Double Digest	Single Digest
Biallelic	Stringently filtered	3170	4015	1731
	Secondary	3481	5133	1404
PAV	Clustered	3374	6738	3202
	Singleton	5342	16937	4015

Table S2. Number of Loci identified by both Single and Double digest GbS libraries in *A. barbata*. The total number of each marker type identified in each library is also given (e.g. out of 8625 biallelic loci in the double digest library, 369 were also identified in the single digest library)

	Double Digest:		
	Biallelic	PAV	Total
Single Digest:			
Biallelic	369	42	2968
PAV	48	731	7217
Total	8652	23675	

Table S3: Statistical significance of the number of homologs/paralogs between pairs of linkage groups. Given the locations of all markers that had a homolog (or paralog in table S3B), the pairings between these markers were randomized 10,000X. For each pair of LGs, the fraction of randomized data sets which exceeded the observed number of homologs for that pair is given. Pairs with < 0.05 (ie occurred significantly more often than expected by chance alone) are highlighted in green.

Table S3A. Homologies between *A. barbata* and *A. strigosa* X *A. wiestii*

	Ab_1	Ab_2	Ab_3	Ab_4	Ab_5	Ab_6	Ab_7	Ab_8	Ab_9	Ab_10	Ab_11	Ab_12	Ab_13	Ab_14
SW_1	0.000	1.000	1.000	1.000	1.000	1.000	1.000	1.000	1.000	0.000	0.764	0.582	0.414	1.000
SW_2	1.000	0.000	1.000	1.000	1.000	1.000	1.000	1.000	1.000	0.999	0.969	0.000	0.379	1.000
SW_3	1.000	1.000	0.000	1.000	1.000	1.000	1.000	0.000	1.000	1.000	0.000	0.971	0.996	1.000
SW_4	1.000	1.000	1.000	0.000	1.000	1.000	1.000	0.994	0.000	0.993	0.952	1.000	1.000	1.000
SW_5	1.000	1.000	1.000	1.000	0.000	1.000	1.000	1.000	0.000	0.000	1.000	0.996	0.935	1.000
SW_6	1.000	1.000	1.000	1.000	1.000	0.000	1.000	0.000	1.000	1.000	0.616	0.750	0.000	0.998
SW_7	1.000	1.000	1.000	1.000	1.000	1.000	0.000	1.000	0.997	1.000	1.000	0.998	0.959	0.000

Table S3B. Paralogs within *A. barbata*

	Ab_1	Ab_2	Ab_3	Ab_4	Ab_5	Ab_6	Ab_7	Ab_8	Ab_9	Ab_10	Ab_11	Ab_12	Ab_13	Ab_14
Ab_1	0.000	0.920	0.999	0.839	0.112	0.897	0.995	1.000	1.000	0.000	0.000	0.985	0.345	1.000
Ab_2		0.000	0.991	0.915	0.290	0.154	0.595	1.000	0.999	1.000	0.949	0.000	0.282	1.000
Ab_3			0.000	0.773	0.952	0.940	1.000	0.000	1.000	1.000	0.086	0.999	0.946	1.000
Ab_4				0.000	0.897	0.797	0.592	1.000	0.000	0.999	0.608	0.973	0.670	0.999
Ab_5					0.008	0.993	0.821	1.000	0.000	0.000	0.989	0.997	0.964	1.000
Ab_6						0.000	0.752	0.000	1.000	1.000	0.381	0.759	0.000	1.000
Ab_7							0.002	1.000	1.000	1.000	0.993	0.998	0.975	0.000
Ab_8								0.000	0.925	0.941	0.476	0.255	0.739	0.483
Ab_9									0.000	0.727	0.407	0.198	0.304	0.935
Ab_10										0.000	0.178	0.224	0.329	0.498
Ab_11											0.002	0.122	0.361	0.046
Ab_12												0.000	0.392	0.630
Ab_13													0.000	0.020
Ab_14														0.000

Table S3C Homologies between *A. barbata* and *A. sativa*

	Ab_1	Ab_2	Ab_3	Ab_4	Ab_5	Ab_6	Ab_7	Ab_8	Ab_9	Ab_10	Ab_11	Ab_12	Ab_13	Ab_14
Mrg1	0.000	1.000	1.000	0.000	1.000	1.000	1.000	1.000	0.154	0.056	0.000	0.577	0.886	0.999
Mrg2	1.000	1.000	1.000	1.000	1.000	1.000	0.000	0.997	1.000	0.996	1.000	1.000	0.978	0.000
Mrg3	0.746	1.000	1.000	0.327	0.028	1.000	0.011	1.000	0.629	0.324	0.391	1.000	1.000	0.634
Mrg4	0.998	0.961	1.000	1.000	0.983	0.000	0.997	1.000	0.891	1.000	1.000	0.655	0.000	0.649
Mrg5	1.000	0.016	0.683	0.994	1.000	0.000	0.999	0.993	0.985	0.872	0.051	0.044	0.000	0.987
Mrg6	1.000	1.000	1.000	0.205	0.000	1.000	1.000	1.000	0.000	1.000	0.690	1.000	1.000	1.000
Mrg8	0.000	0.000	1.000	0.997	1.000	1.000	1.000	0.996	0.992	0.000	1.000	0.000	0.014	0.864
Mrg9	0.823	1.000	0.987	1.000	0.000	0.964	1.000	1.000	0.290	0.008	1.000	1.000	1.000	1.000
Mrg11	1.000	1.000	0.000	0.401	1.000	0.176	0.999	1.000	0.854	1.000	0.000	0.603	1.000	0.588
Mrg12	0.000	0.999	1.000	1.000	1.000	1.000	0.000	1.000	0.999	1.000	0.029	0.973	0.988	0.000
Mrg13	1.000	0.000	1.000	1.000	1.000	0.965	1.000	0.833	0.665	0.733	0.421	0.002	1.000	1.000
Mrg15	1.000	1.000	0.000	1.000	1.000	0.940	1.000	0.000	1.000	0.663	1.000	1.000	1.000	1.000
Mrg17	0.894	0.278	0.995	1.000	1.000	0.516	0.000	1.000	1.000	0.789	0.483	1.000	0.000	0.737
Mrg18	0.000	1.000	1.000	1.000	1.000	1.000	1.000	1.000	1.000	0.000	0.842	1.000	0.514	0.999
Mrg19	0.005	1.000	0.000	0.847	0.999	0.991	1.000	0.227	1.000	1.000	1.000	0.037	1.000	1.000
Mrg20	1.000	1.000	1.000	0.006	0.000	0.000	1.000	0.000	0.960	0.281	1.000	1.000	1.000	1.000
Mrg21	1.000	1.000	1.000	1.000	0.000	0.004	0.572	0.000	0.971	0.054	1.000	1.000	1.000	0.546
Mrg23	1.000	1.000	0.000	1.000	1.000	1.000	1.000	0.179	1.000	1.000	0.950	0.988	1.000	1.000
Mrg24	1.000	0.995	1.000	0.000	0.000	1.000	1.000	1.000	0.000	0.995	0.832	1.000	1.000	0.988
Mrg28	0.825	1.000	0.000	1.000	1.000	0.999	1.000	0.000	1.000	1.000	0.778	1.000	1.000	1.000
Mrg33	1.000	0.000	1.000	0.998	1.000	1.000	0.000	1.000	1.000	1.000	1.000	0.003	1.000	0.000

Table S3D Homologies between *A. sativa* and *A. strigosa* X *A. wiestii*

	SW_1	SW_2	SW_3	SW_4	SW_5	SW_6	SW_7
Mrg1	0.000	0.998	1.000	0.044	1.000	0.998	1.000
Mrg2	1.000	0.943	1.000	1.000	1.000	1.000	0.000
Mrg3	0.934	1.000	0.808	1.000	0.007	1.000	0.410
Mrg4	1.000	0.904	1.000	0.751	0.982	0.000	0.861
Mrg5	1.000	0.177	0.931	0.951	1.000	0.000	0.998
Mrg6	1.000	1.000	1.000	0.003	0.000	1.000	1.000
Mrg8	0.000	0.000	1.000	0.926	1.000	1.000	0.999
Mrg9	0.977	1.000	0.642	0.441	0.000	1.000	1.000
Mrg11	0.999	1.000	0.000	0.672	0.999	0.125	0.982
Mrg12	0.000	0.982	1.000	0.990	1.000	1.000	0.000
Mrg13	1.000	0.000	1.000	1.000	0.923	1.000	0.179
Mrg15	1.000	1.000	0.000	1.000	0.847	1.000	1.000
Mrg17	0.722	1.000	1.000	1.000	1.000	0.437	0.000
Mrg18	0.000	1.000	1.000	1.000	1.000	1.000	1.000
Mrg19	0.028	1.000	0.002	1.000	0.979	1.000	1.000
Mrg20	1.000	1.000	0.994	0.032	0.000	0.000	1.000
Mrg21	1.000	0.999	1.000	1.000	0.000	0.001	0.423
Mrg23	1.000	0.999	0.000	0.932	1.000	1.000	1.000
Mrg24	1.000	0.999	1.000	0.000	0.000	1.000	1.000
Mrg28	0.996	1.000	0.000	1.000	1.000	0.999	0.968
Mrg33	1.000	0.000	1.000	1.000	1.000	0.986	0.000

Supplementary Figures

Figure S1. Single vs Double restriction digest GBS linkage maps in *A. barbata*. The linkage map presented in the text (Fig. 2) used the double digest GBS library preparation protocol of Poland et al. [42]. The linkage map from the single digest GBS protocol[43] gave a closely similar map. For each LG, the double digest map is shown on the left while the single digest map is on the right. Loci detected by both protocols were relatively rare (Supplemental Table S2), but reveal a strong similarity between the two maps (Blue diamonds with connector lines).

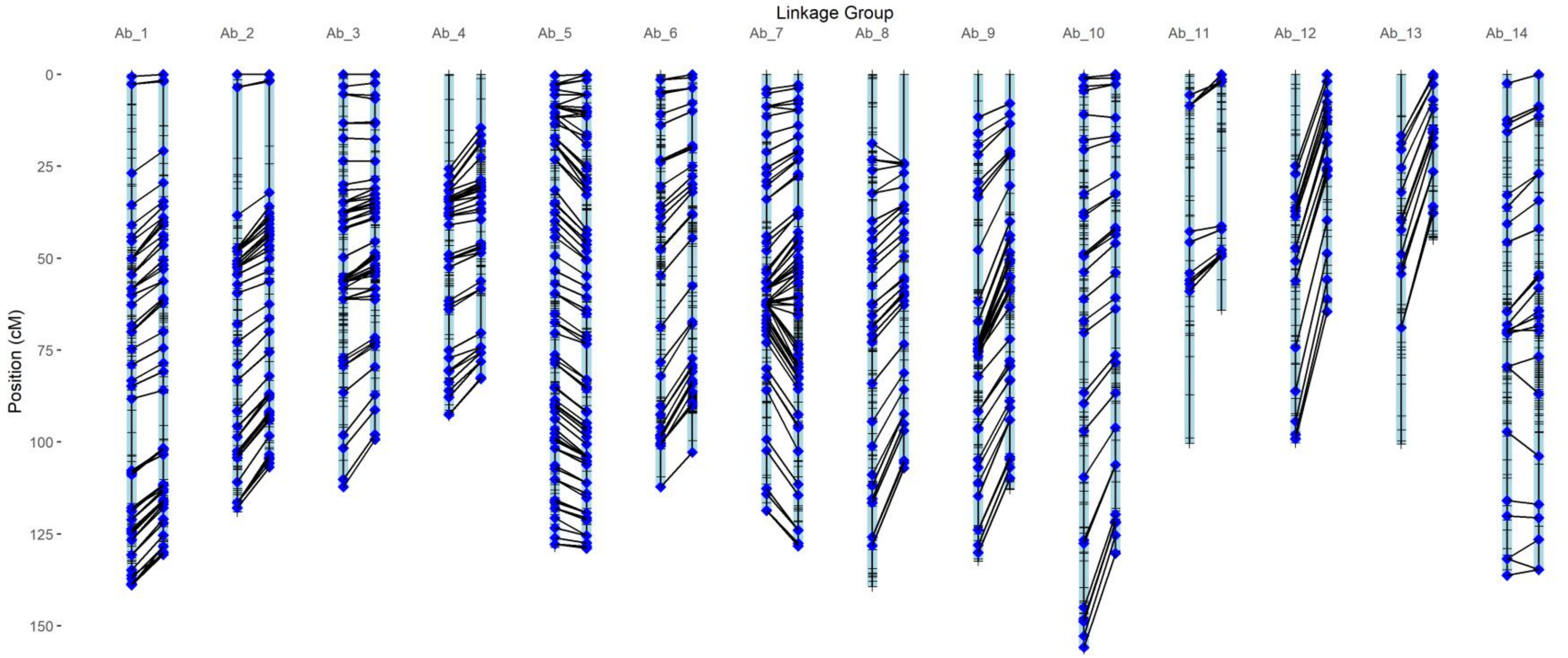

Figure S2 Hypothesized possible evolutionary transition between A and B genome karyotypes.

Panel A (this page). Homeologous regions of the A and B genome of *A. barbata*, redrawn from Fig 4b of the main text. Linkage groups are coloured red for the A genome and blue for the B genome. Nine translocated blocks are labelled A-I. Note the similarity to Fig. 2 of Udall et al [7], and Fig. 1a of Cheng et al, [12] in *Brassica napus*. Panel B (next page). One possible sequence of translocations rearranging the A_s genome of *A. strigosa* and *A. wiestii* to the B genome of *A. barbata*. Three reciprocal translocations followed by one non reciprocal translocation (technically a reciprocal translocation where one of the exchanged fragments is very small, *cf* Schubert and Lysak, [57]) which breaks a cyclical translocation into what we've called an enchained translocation. Chromosomal blocks are labelled following Fig. S2a and colour coded by linkage group of the A genome. Intermediate karyotypes are labeled X, Y and Z, and bold lettering indicates where the B genome chromosomes first appear in this sequence. Note however, that since any of these translocations could occur in either direction, any of these karyotypes could be ancestral. Note too that the linkage group Ab-5′ resembles the arrangement seen in Mrg_20 (A genome) and Mrg_21 (D genome) of *A. sativa*, and we could speculate origin of those chromosomes from a karyotype similar to the "X –genome".

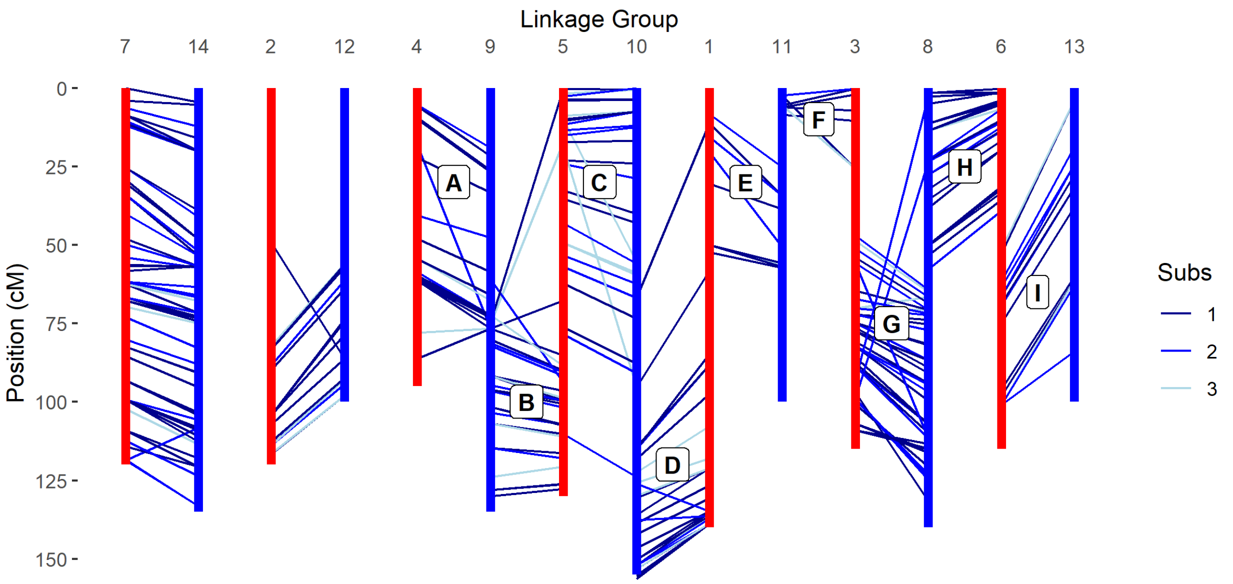

Fig S2B (Caption on prev page)

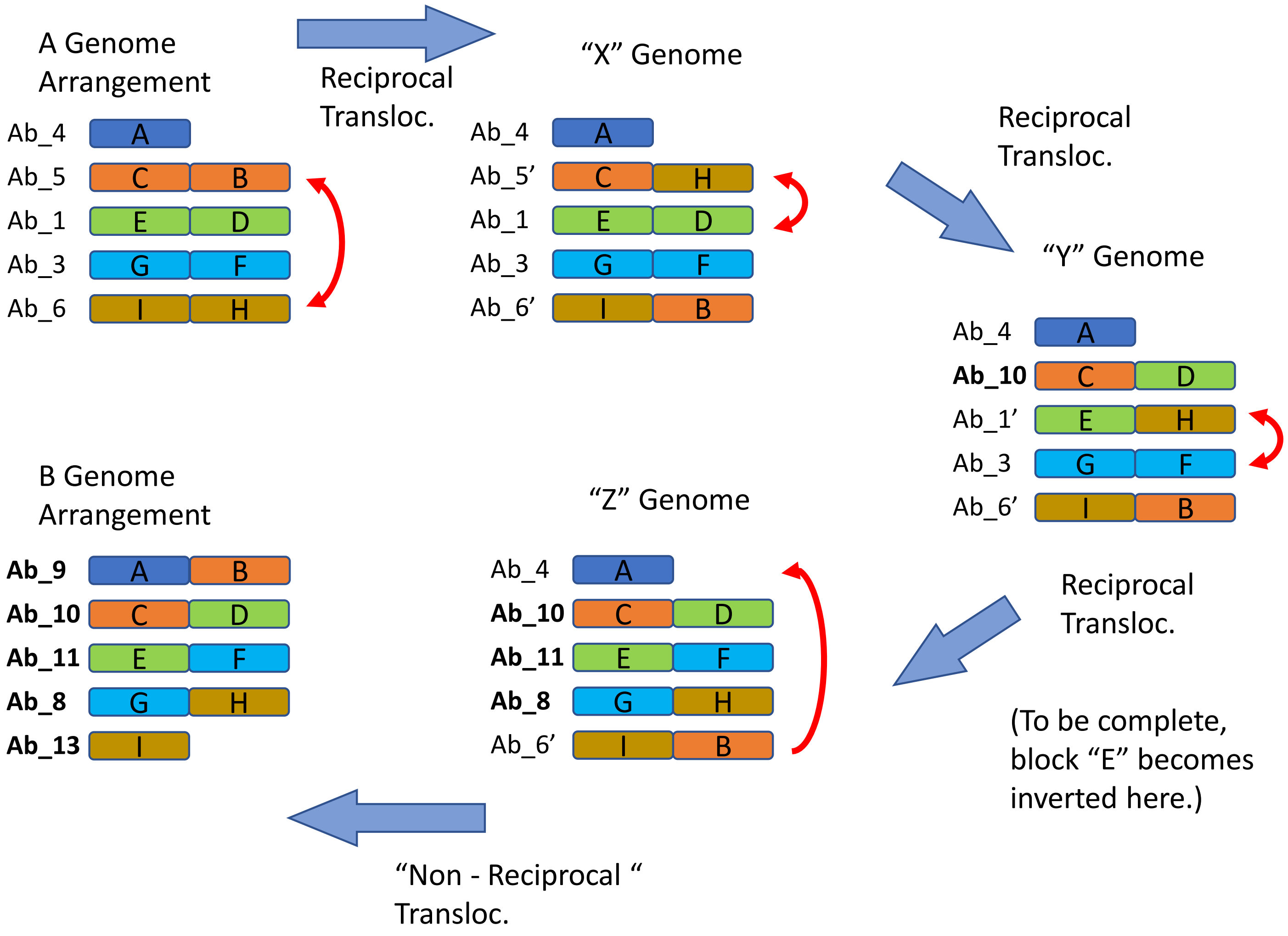

Supplement: Supplementary file 2 — Supplementary Info [file 41598_2019_48639_MOESM2_ESM.pdf]
